# Feasibility study to assess the delivery of a novel isometric exercise intervention for people with stage 1 hypertension in the NHS: protocol for the IsoFIT-BP study including amendments to mitigate the risk of COVID-19

**DOI:** 10.1186/s40814-021-00925-w

**Published:** 2021-10-28

**Authors:** Jonathan Wiles, Melanie Rees-Roberts, Jamie M. O’Driscoll, Timothy Doulton, Douglas MacInnes, Vanessa Short, Tracy Pellatt-Higgins, Katie Saxby, Katerina Gousia, Alan West, Maggie Smith, Ellie Santer, John Darby, Chris K. Farmer

**Affiliations:** 1 Faculty of Science, Engineering and Social Sciences, Canterbury Christ Church University, Canterbury, Kent UK; 2 Centre for Health Services Studies, University of Kent, Canterbury, Kent UK; 3 Renal Department East Kent Hospitals University NHS Foundation Trust, Canterbury, Kent UK; 4 Faculty of Medicine, Health and Social Care, Canterbury Christ Church University, Canterbury, Kent UK; 5 Kent, UK

**Keywords:** Isometric exercise, Exercise, Hypertension, General practice, Feasibility study, COVID-19

## Abstract

**Background:**

Hypertension  (HTN) affects approximately 25% of the UK population and is a leading cause of mortality. Associated annual health care costs run into billions. National treatment guidance includes initial lifestyle advice, followed by anti-hypertensive medication if blood pressure (BP) remains high. However, adoption and adherence to recommended exercise guidelines, dietary advice and anti-hypertensive medication is poor. Four short bouts of isometric exercise (IE) performed 3 days per week (d/wk) at home elicits clinically significant reductions in BP in those with normal to high-normal BP. This study will determine the feasibility of delivering personalised IE to patients with stage 1 hypertension for whom lifestyle changes would be recommended before medication within NHS primary care.

**Methods:**

This is a randomised controlled feasibility study. Participants were 18+ years, with stage 1 hypertension, not on anti-hypertensive medication and without significant medical contraindications. Trial arms will be standard lifestyle advice (control) or isometric wall squat exercise and standard lifestyle advice. Primary outcomes include the feasibility of healthcare professionals to deliver isometric exercise prescriptions in a primary care NHS setting and estimation of the variance of change in systolic BP. Secondary outcomes include accuracy of protocol delivery, execution of and adherence to protocol, recruitment rate, attrition, perception of intervention viability, cost, participant experience and accuracy of home BP. The study will last 18 months. Sample size of 100 participants (50 per arm) allows for 20% attrition and 6.5% incomplete data, based upon 74 (37 each arm) participants (two-sided 95% confidence interval, width of 1.33 and standard deviation of 4) completing 4 weeks. Ethical approval IRAS ID is 274676.

**Discussion:**

Before the efficacy of this novel intervention to treat stage 1 hypertension can be investigated in any large randomised controlled trial, it is necessary to ascertain if it can be delivered and carried out in a NHS primary care setting. Findings could support IE viability as a prophylactic/alternative treatment option.

**Trial registration:**

ISRCTN13472393, registered 18 August 2020

**Supplementary Information:**

The online version contains supplementary material available at 10.1186/s40814-021-00925-w.

## Introduction

### Background and rationale

Hypertension (≥ 140/90 mmHg) [1] affects approximately 1 in 4 people in the UK and is a leading modifiable risk factor for mortality [2]. As the most common long-term health condition in the UK and a primary risk factor for mortality [2], hypertension is a serious health problem [3, 4]. With every 20 mmHg increase in systolic BP above 115 mmHg and 10 mmHg increase in diastolic BP above 75 mmHg, the risk of mortality from cardiovascular disease doubles [5]. Public Health England (PHE) suggests that there is an opportunity to prevent more than 9000 heart attacks and at least 14,000 strokes over 3 years with better detection and management of high BP, high cholesterol and atrial fibrillation [6]. Estimates indicate that the annual burden from conditions attributable to hypertension is over £2 billion in England [4], with long-term care following a debilitating heart attack, stroke and/or vascular dementia often precipitated by hypertension costing substantially more [7, 8]. It has been suggested that over ten years, 45,000 quality adjusted life years and £850m could be saved if England achieved a 5 mmHg reduction in population systolic BP [4, 8]. In 2007, the NHS and PHE announced a drive to prevent thousands of heart attacks and strokes but highlighted the need for research into innovative lifestyle modifications to lower BP [4]. New personalised support for lifestyle changes [3], like our IE training prescription [9, 10], delivered by primary care allied health professionals could support this drive without directly adding to GP workload [11].

#### Standard care for hypertension

National guidance for the treatment of hypertension is graded from lifestyle intervention (i.e. advice about diet, weight management, exercise, alcohol intake) to pharmacological therapy depending upon severity and duration of hypertension when other factors have been taken into account such as age, co-morbidity and the presence of target organ damage [1, 12]. The goal of antihypertensive therapy is generally to reduce clinic BP to < 140/90 mmHg, although recommended targets vary depending on age and co-morbidity [1, 13]. However, up to 50% of people fail to achieve their target BP [14, 15] mainly due to non-compliance (estimated 30–50% failing to comply at 6 and 12 months, respectively) [16, 17] with undesirable side effects of anti-hypertensive medication often cited in this context [18, 19].

#### Lifestyle interventions

The importance of lifestyle changes to aspects, such as diet and exercise habits for patients with hypertension in the absence of other risk factors should not be overlooked [1, 20]. Additional treatment options are also in keeping with the UK government’s commitment to provide greater patient choice [21]. Whilst dietary strategies can be effective, these are difficult to fully adopt and maintain [22, 23].

Evidence suggests that exercise may be as effective as medication in controlling BP [24–26] and it is often promoted as a treatment option for those with stage 1 hypertension (defined as a blood pressure of 140–159/90–99 mmHg), without co-morbidity or other long-term conditions [1, 27]. Current exercise guidelines for the prevention and treatment of hypertension recommend that adults accumulate a mainstay of 30+ min of moderate intensity aerobic exercise on 5, but preferably all, days per week (d/wk), supplemented by moderate to vigorous resistance exercise 2–3 d/wk and general flexibility exercise ≥ 2–3 d/wk [28, 29]. However, in a randomised controlled trial, a long-term programme of aerobic exercise training failed to reduce clinic BP [30]. This may be due to poor adherence (e.g. 67%) to the relatively high amounts of aerobic exercise recommended [30, 31]. Furthermore, other studies have demonstrated attrition rates as high as 50% during traditional aerobic exercise interventions [32, 33]. Indeed, evidence typically suggests that a significant challenge is the low adoption and high attrition rates associated with these guidelines [34]. Thus, effective and manageable lifestyle interventions with respect to exercise remain an unmet clinical need [3]. It is suggested that to promote lifestyle exercise changes, patients need easily adopted, effective and manageable exercise interventions as a first line option for managing their BP.

#### Isometric exercise

**Fig. 1:**
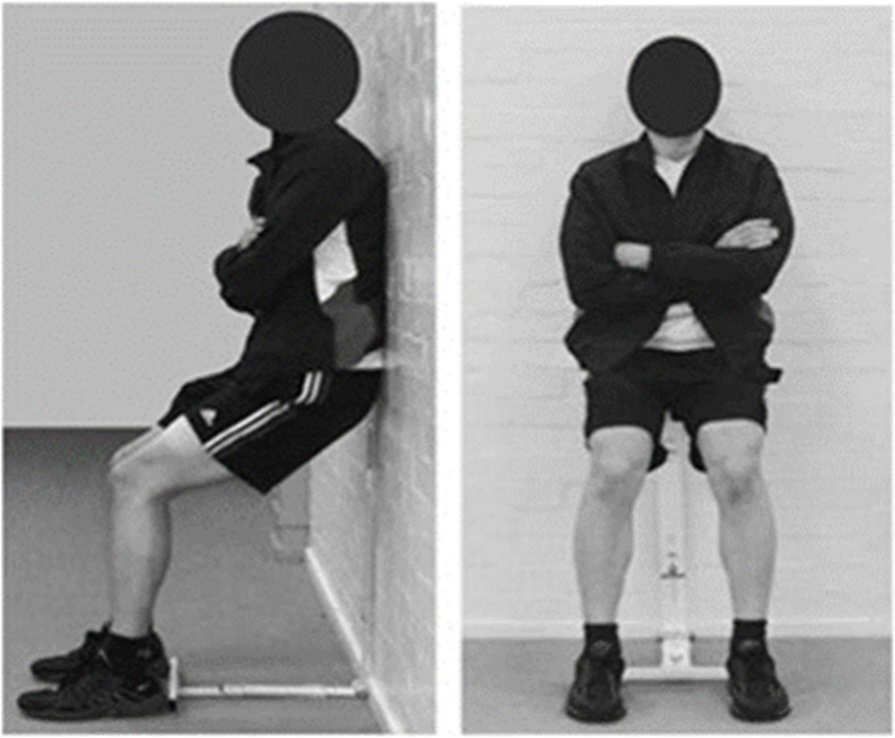
Isometric wall squat exercise

Meta-analyses indicate that isometric exercise (IE) results in larger reductions in BP when compared with either aerobic and dynamic resistance exercise training [25, 35] and has great potential to treat hypertension [10, 36] and improve cardiovascular health, which may reduce mortality risk [37]. We have shown that IE can lower BP in people with both normal [9, 38] and high-normal (pre-hypertensive) [10, 39] BP and therefore has the potential to be an effective lifestyle intervention for hypertension. IE involves holding a fixed position for a period of time; skeletal muscles are used but there is no movement, e.g. the wall squat which involves leaning against a wall in a seated position (Fig. 1).

Evidence suggests that IE offers distinct advantages over other forms of exercise [10, 36] making it a better lifestyle treatment for hypertension. A frequently cited barrier to exercise is lack of time [40, 41]. Though aerobic exercise guidelines recommend ≥ 150 min/week [42], it has been demonstrated that adherence is better with shorter bouts of exercise [43, 44]. Only 24 min of isometric wall squat exercise a week are required to achieve clinically significant reductions in BP of 12/6 mmHg in pre-hypertensives [10]. Moreover, our evidence-based IE wall squat programme can be easily prescribed by health care providers and is very simple to execute regardless of age or physical ability [10, 45]. It does not require costly equipment or access to specialist facilities, does not require specific clothing, and most importantly, is easily performed at home [9, 10]. Empowering patients to manage their condition is key and use of short, simple, personalised exercise that can be carried out at home may enable this [46, 47]. Furthermore, our personalised exercise ‘prescription’ ensures optimal IE intensity is achieved, helping to improve confidence, motivation and patient adherence [40, 48].

### Contextualising current findings

The importance of these findings is substantial [49] considering a 10 mmHg reduction in systolic BP and 5-mmHg reduction in diastolic BP is associated with a 40% lower risk of stroke and 30% lower risk of mortality from heart disease and other vascular causes throughout middle age [6]. Although we have consistently demonstrated that IE can lower BP [10, 37, 38, 50], interpretation of the results, along with the findings of others is limited by small participant numbers [9, 10, 37, 51–56]. Isometric exercise may provide a new viable solution with respect to exercise for those with stage 1 hypertension, but evidence for the efficacy of IE in this clinical population is still not robust. Furthermore, this intervention has never been tested within a NHS setting, nor confirmed in any large randomised control trials. This feasibility study was deemed necessary by the NIHR to determine uncertain parameters necessary to inform and justify the design and further funding of a large scale randomised controlled trial to evaluate the efficacy and mechanisms of isometric wall squat exercise to lower BP in stage 1 hypertensive NHS patients.

## Objectives

The aim of the study is to determine the feasibility of delivering an individually tailored IE training programme to patients with stage 1 hypertension (defined as a clinic BP of 140–159/90–99 mmHg) for whom lifestyle changes would be recommended before pharmacological treatment within a primary care NHS setting. Furthermore, the primary objectives are as follows:

1. To assess if healthcare professionals (e.g. nurses, health trainer, healthcare assistants, physiotherapists) can deliver isometric exercise prescriptions for stage 1 hypertensive patients in a primary care NHS setting
2. To estimate the variance in BP change, to enable sample size calculation for a definitive randomised controlled trial

The secondary objectives are as follows:

1. Evidence the fidelity of the study intervention with respect to healthcare professional delivery and patient completion of IE
2. Estimate short- (4 week) and medium-term (3 and 6 month) adherence rates to IE intervention
3. Estimate recruitment and attrition rates at recruiting GP sites to inform future trials
4. Explore the willingness of GPs, secondary care clinicians and healthcare professionals to consider IE as a treatment option for patients, including barriers and facilitators for delivering and integrating this within an NHS care pathway for hypertension
5. Establish the cost and cost-utility of the IE intervention compared to standard care for stage 1 hypertension
6. Understand participant experiences of undertaking IE, adherence to the programme and continuation. Additionally, explore possible negative effects of COVID-19 on recruitment rates and participation
7. To investigate the feasibility of using observed home blood pressure readings for remote monitoring

## Trial design

### Design

Multi-centre randomised controlled feasibility study.

### Random allocation

Those that meet the inclusion criteria will be randomised to one of two groups in a 1:1 ratio. One hundred participants will be recruited; therefore, 50 participants will be allocated to each group.

### Amendments due to COVID-19 pandemic

As a result of the unprecedented spread of a novel coronavirus early in 2020, by mid-March the European region had become the epicentre of a COVID-19 pandemic [57]. An outcome of this was that many recently funded health projects using human participants, including this study, were required to re-evaluate their viability and where necessary revise their research design, methods and protocols in line with general Government and specific funder guidance (e.g. NIHR Restart Framework [58]) to help ensure the continued safety of research participants and personnel alike. The main changes made to this study are documented (*identified in italics*) to provide the reader with greater insight into how this research project evolved to become fit for purpose as part of what has now become a new research normal.

## Methods: participants, interventions and outcomes

### Study setting

GP practices in the South East of England.

### Eligibility criteria

Patients aged 18+ with stage 1 hypertension who are not yet on anti-hypertensive medication and without any significant medical condition that would contraindicate their participation.

### Exclusion criteria

Patients who are taking anti-hypertensive medication; have white coat hypertension (as evidenced by averaged home systolic BP < 135 mmHg); are unable to undertake the study intervention (isometric exercise); have a previous history of diabetes mellitus (type 1 or type 2), known or suspected ischaemic heart disease (including myocardial infarction and/or angina and/or coronary revascularization procedure), moderate or severe stenotic or regurgitant heart valve disease, atrial or ventricular arrhythmia, stroke or transient ischaemic attack, aortic aneurysm and/or peripheral arterial disease, uncorrected congenital or inherited heart condition; have an estimated glomerular filtration rate < 45 ml/min (calculated using CKD-EPI or MDRD formulae, and taking most recent documented results); have a documented left ventricular ejection fraction < 45% and/or left ventricular hypertrophy (by either echocardiography or standard ECG criteria, e.g. Sokolow-Lyon); have a documented urine albumin to creatinine ratio > 3.5 mg/mmol; are unable to provide informed consent; are enrolled in another Clinical Trial of an Interventional Medicinal Product or Medical Device or other interventional study; and if female, are pregnant or currently breast feeding. Then finally, any medical condition that, in the opinion of the investigator, would make the participant unsuitable for the study.

### Who will take informed consent?

Trained healthcare professionals will be obtaining informed consent via discussion on *video call and completion via online software* or hardcopy.

## Interventions

### Explanation for the choice of comparators

National guidance for patients newly diagnosed with stage 1 hypertension is two tiered and recommends that GP’s normally provide advice about potential lifestyle (non-pharmaceutical) changes that may help to reduce BP in the first instance before prescribing antihypertensive medication [1]. As such, the comparator used in this study will be defined as ‘standard care lifestyle advice’. One potential issue raised during preliminary consultation with PPI members, the healthcare professionals (including GP’s) in primary and consultants in secondary care was related to the concept of standard care lifestyle advice. It quickly became evident that there are differences in the quality and consistency of standard care lifestyle advice provided by primary care centres across the region (and indeed the UK). As such, it was agreed by the Project Management Group that for accurate comparison of the intervention (arm 1) with the control (arm 2), standard care lifestyle advice would need to be as consistent as possible across participating sites. The best way to achieve this within the project time frame was to adhere to common published guidelines that would be given to all HCP’s and patients involved. As such, the project adopted an existing peer reviewed leaflet produced by the East Kent Hospitals University NHS Foundation Trust (EKHUFT) entitled: High Blood Pressure (Hypertension) - Information for Patients from the Department of Renal (Kidney) Medicine.

### Intervention description

**Fig. 2:**
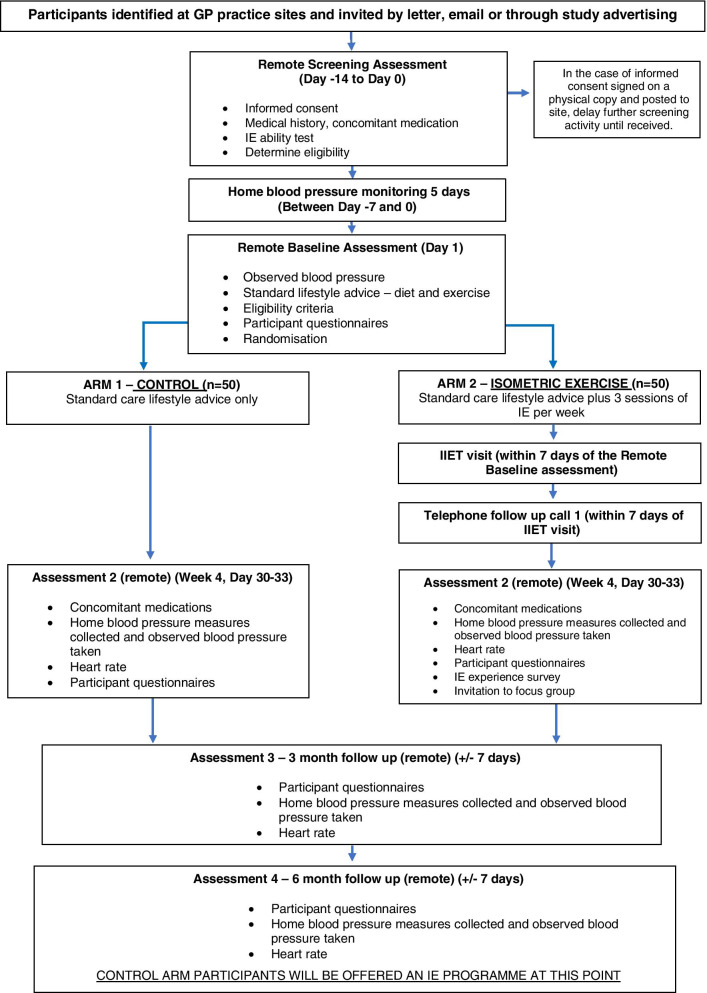
Trial flow chart

Standard care lifestyle advice (arm 1 — control) or isometric wall squat exercise training with standard care lifestyle advice (arm 2). Figure 2 shows flow-chart of selection of participants and interventions.

The following lifestyle information will be provided to all participants taking part in the study.

1. Lifestyle changes that are recommended for people with high blood pressure include the following:
  - Salt intake. The average salt intake in the UK is 9 g/day. The recommended daily salt intake for an adult is 5 to 6 g/day. Reducing the amount of salt in your food lowers blood pressure and makes blood pressure lowering medications more effective. Although stopping adding salt to your food when cooking or at the table is important, 80% of the salt we eat is already present in food when we buy it — so called ‘hidden salt’. Understanding food labelling can help you see where these hidden sources of salt are found.
  - Healthy diet. You should follow a diet that is rich in fresh fruit and vegetables and low in saturated fat.
  - Weight. You should try to maintain a body mass index between 20 and 25 kg/m^2^. Those with BMI greater than 30 kg/m^2^ and high blood pressure should definitely try to lose weight to achieve a BMI of 30 kg/m^2^ or below. See BMI chart at the end of this leaflet.
  - Alcohol consumption. You should adhere to the current UK limit of less than 14 units per week. If you do regularly drink as much as 14 units in a week try to spread your drinking evenly over three days or more. One unit of alcohol is equivalent to half-pint of average strength beer, a small glass of wine (125 ml), or a single pub measure of spirits. Remember that stronger beers (such as continental lager) and larger glasses of wine (175 ml or 250 ml) will contain more units.
  - Exercise. You should try to exercise at a level that makes you breathless for at least 30 min three times a week, although more is recommended if possible. A variety of different types of exercise — aerobic/endurance (for example running) and resistance (for example weights) — both appear to be equally helpful in reducing blood pressure.
2. For more information on lifestyle modification visit the Blood Pressure UK website:
  - www.bloodpressureuk.org/BloodPressureandyou/Yourlifestyle

The full leaflet ‘High Blood Pressure (Hypertension) - Information for Patients from the Department of Renal (Kidney) Medicine’ is presented as an additional file (see Additional file 1).

**Fig. 3:**
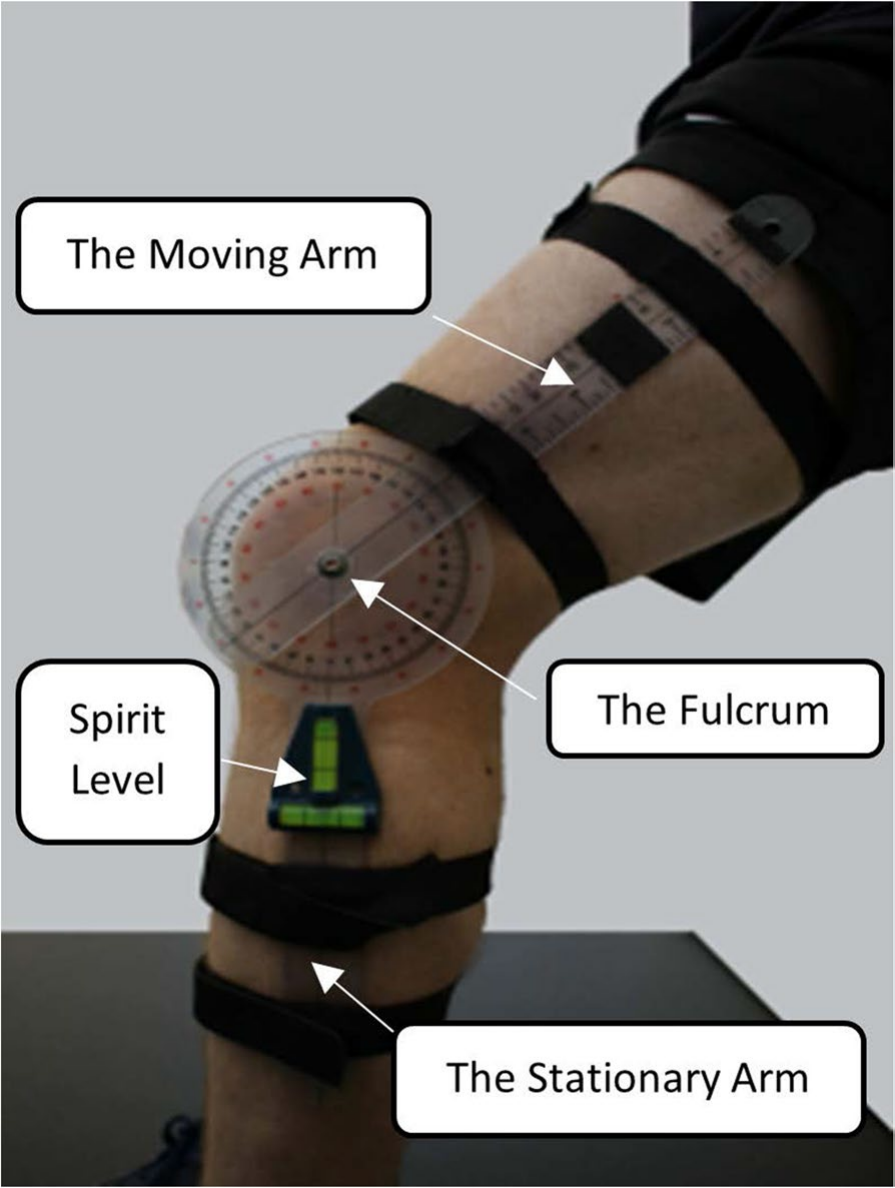
Goniometry of the knee

Participants allocated to the intervention arm 2 will be required to attend their GP surgery to perform an incremental isometric wall squat test. This test will be delivered by the designated healthcare professional (HCP) previously trained in the delivery of this protocol. Upon arrival participants will be familiarised with a simple rating of perceived exertion scale validated specifically for use with isometric exercise [59]. Following this, they will be fitted with a wireless heart rate monitor chest strap and wrist watch (Sigma PC 15.11, Neustadt/Weinstraße, Germany) and MIE clinical goniometer (MIE Medical Research Ltd., Leeds, UK), which will be attached to the participant’s left leg using elastic Velcro straps. The fulcrum will be aligned with the lateral epicondyle of the femur, and the moving arm is placed on the lateral midline of the femur using the greater trochanter for reference and the stationary arm on the lateral midline of the fibula using the lateral malleolus and fibular head for reference. A spirit level will be attached to the stationary arm to ensure that the lower leg is kept vertical during exercise (Fig. 3) [37].

The participant will then be asked to perform a continuous isometric wall squat test in stages of increasing intensity, which is determined by manipulating knee joint angle with the internal angle between the femur and fibula measured. Based upon the work of Goldring et al. [53], the first stage begins at 135° of knee flexion, and participants are instructed to hold this position for 2 min. HR is measured continuously throughout the incremental test with HR recorded every 5 s during the last 30–0 s of each 2-min increment. Wall (measured as floor to coccyx height) and floor (measured as wall to back of the heels) positions are also recorded (cm) in the final 10 s of each increment. At the end of each increment, participants will be asked to rate their perceived exertion to give the healthcare professional an indication of how close they are to finishing the test. Once each stage is complete the knee joint angle will be decreased by 10°. The angle is then decreased every 2 min until the participant reaches the end of the 95° stage or can no longer maintain the knee joint angle within 5° of the target value (volitional fatigue). The HCP’s were instructed to maintain a good dialogue (providing as much encouragement as was necessary) with the participant throughout, to ensure they stay calm and relax their breathing to avoid the Valsalva manoeuvre, to watch closely for any signs of physical distress and not to hesitate to stop the test immediately if they feel the participant’s health is at risk [60].

Based upon each participant’s test data, knee joint angle is plotted against the mean HR for the last 30 s of each incremental stage. Since an accurate IE wall squat prescription requires data from at least 3 complete stages, any participant unable to achieve this will be classed as a screen failure and informed that they will not be able to take part in the study. The relationship between parameters is then used to calculate the specific knee joint angle required to elicit a target HR. The target HR selected for training is 95% HR_peak_, with HR_peak_ defined as the mean HR of the final 30 s achieved during the incremental test [60]. Additionally, the individual target heart rate range (THRR) will be established using the 95% reference interval [61].

During the 10-min data analysis period (performed remotely by the study research assistant), the patient will be taken through the participant information and their personal IE training diary (used to record the HR and RPE data from each training session performed in the home) to ensure that they fully understand what is required of them throughout the 6-month training period. At this stage, the patient will also be issued with their own HR monitor, shown how to work it and given a written instruction sheet for future reference.

**Fig. 4:**
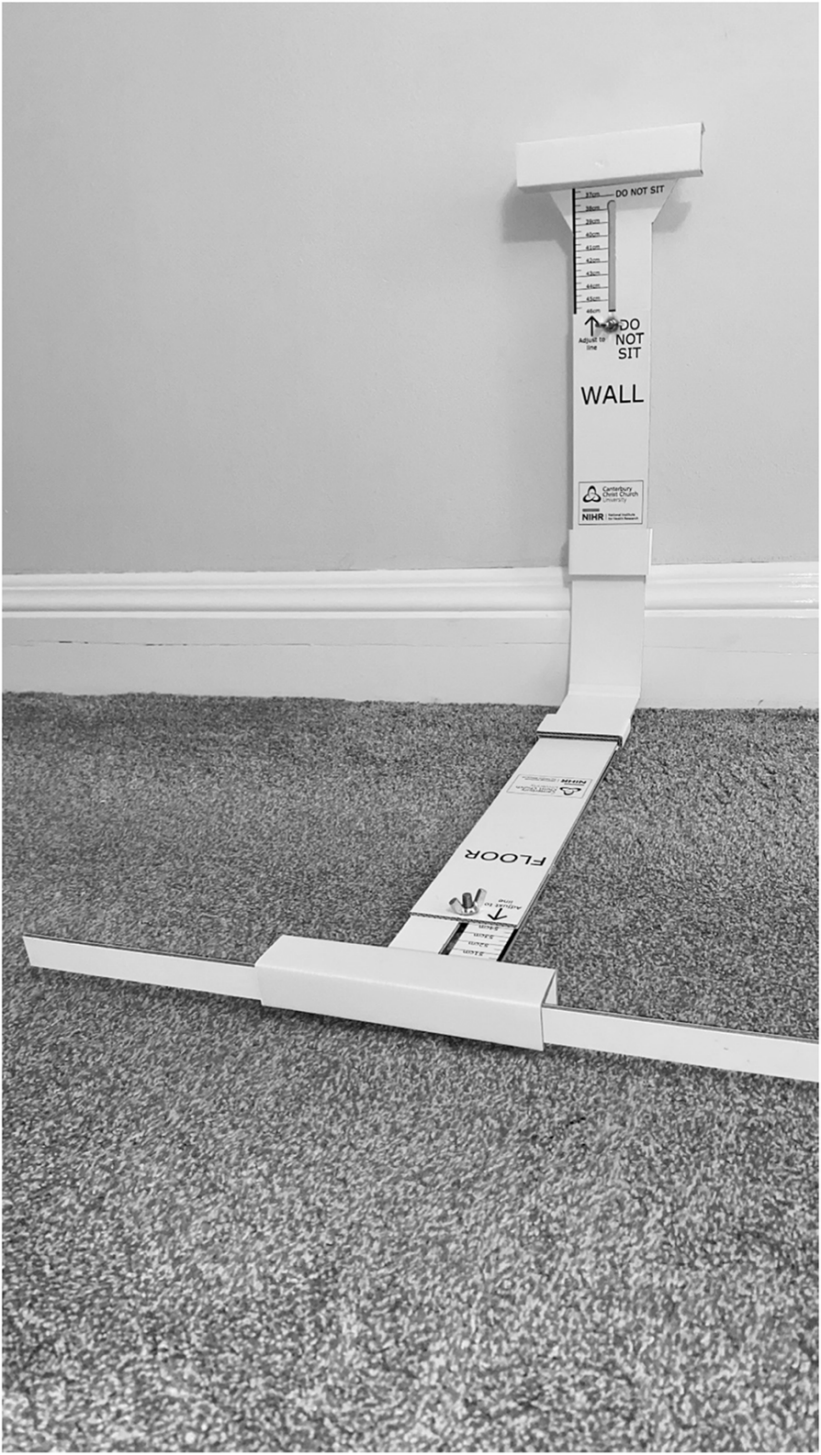
Bend and Squat device used to determine knee joint angle during wall squat exercise

A goniometer is not deemed practical for participant measurement of knee joint angle during home-based training; therefore, a simple device called the ‘Bend and Squat’ is used to align a participant’s feet and back into the correct position for a given wall squat knee joint angle (Fig. 4) [55].

Once the device is set to the participants personal wall and floor measurements necessary to replicate the angle required to elicit 95% HR_peak_, they will then be asked to squat in this set position as a fidelity check to ensure that it equates to their specific training knee joint angle. In the unlikely event that it is necessary, a slight adjustment to the wall height can be used to achieve the correct knee joint angle prescribed. Before the client leaves the surgery to commence their training, it will be ensured that they are completely confident with the information they have received. *Participants will then given a web link (USB stick if required) containing video instructions of how to set up their HR monitor, use the Bend and Squat device to perform a IE training session and how to record the necessary training data in their diaries*. They will also be provided with the study research assistant contact telephone and e-mail helpline address for use during office hours.

All IE training sessions thereafter will be completed in the home. Participants will use their Bend and Squat device to perform an IE training session composed of 4 bouts of 2-min wall squats with 2 min recovery in between each bout [60]. Participants will be instructed to perform three IE training sessions a week, ideally on alternate days to allow for adequate between session recovery. During the IE training sessions, the HR value displayed at the end of each 4 × 2-min wall squat along with their RPE will be recorded for subsequent analysis. If the mean HR of the four exercise bouts deviates from the prescribed THRR on two consecutive sessions in the first week of IE training (acting as a fidelity check), the knee joint angle and thus their Bend and Squat set-up will be altered accordingly based upon further interpolation of their HR/knee joint angle relationship.

### Criteria for discontinuing or modifying allocated interventions

Modification of the isometric exercise prescription will be made if appropriate, after the participant’s first week of exercise training, as part of the IE protocol fidelity assessment.

### Strategies to improve adherence to interventions

Participants will receive reminder text or email messages to help adherence to standard care advice collecting home blood pressure measurements and isometric exercise training.

A bespoke automated reminder system was developed in order to manage the different types of reminders and different periodicity of these.

Arm 1 (control) will receive monthly reminders to adhere to the standard care lifestyle advice given by their HCP at Baseline. Arm 2 (intervention) will also receive monthly standard care lifestyle advice in addition to three IE training reminders per week, for the duration of the study.

At follow up timepoints 4 weeks, 3 months and 6 months, all participants will receive a 24-h reminder to start taking their home blood pressure.

### Relevant concomitant care permitted or prohibited during the trial

Exclusion criteria states the participants should not be taking anti-hypertensive medications whilst taking part in the trial.

### Provisions for post-trial care

The participants in the control arm will be offered an isometric exercise intervention after they have completed their time in the study. The GP practice will oversee participant’s general care throughout the trial and participants will be returned to the care of their GP after the trial.

### Outcomes

#### Primary

1. Feasibility of isometric exercise prescription assessed using qualitative data from healthcare professional focus groups at month 11/12 of 18 month study
2. Feasibility of isometric exercise intervention and study assessed using qualitative data from participants at month 7 and 11 of 18 month study
3. Variance of blood pressure changes from baseline using participant blood pressure data at week 4 and months 3 and 6 of intervention
4. Sample size for a definitive randomised controlled trial, calculated using estimate of variability of evidence of effect on systolic blood pressure change at week 1 and months 3 and 6 (of the intervention) from the feasibility study and the minimum clinically important difference
5. Estimate of the treatment difference in systolic BP change from baseline and 80 and 95% confidence intervals to provide preliminary assessment of benefit, and aid decision whether to proceed to a confirmatory RCT at the end of the feasibility study

#### Secondary

1. Fidelity of the isometric exercise prescription measured using prescription competency assessment data (ability to carry out incremental isometric exercise test and an accurate prescription of individual IE intensity - knee joint angle translated into bend and squat measurements) from HCP intervention training at month 3 of 18 month study
2. Fidelity of the delivery of the isometric exercise prescription by HCP assessed through observation (study coordinator) of the first Incremental Isometric Exercise Test (IIET) and subsequent prescription delivered at site from month 3 to month 5 of 18 month study
3. Fidelity of the isometric exercise prescription defined by participant 95% peak heart rate falls within their THRR in at least two-thirds of all training sessions at say 7–10 of intervention
4. Short and medium-term adherence rates recorded as those adhering to isometric exercise intervention at week 4, month 3 and month 6 of intervention

5. Recruitment and attrition rates from data collected at sites at month 10 and month 15 of 18 month study
6. GPs and healthcare professionals’ attitudes to isometric exercise as a treatment option for patients, measured using remote focus groups and telephone interviews at month 11/12 of 18 month study
7. Cost and cost-utility of the isometric exercise intervention using healthcare resource use data and quality-adjusted life years (QALYs) at month 15 (or last patient follow up) of 18 month study
8. Participant experiences of undertaking isometric exercise using participant isometric exercise experience surveys at week 4 of intervention
9. *Effect of COVID-19 on recruitment rates and participation using participant focus groups or telephone calls at month 7 and 11 of 18 month study*
10. *Feasibility of using observed home blood pressure readings for remote blood pressure monitoring, using participant blood pressure data and observations from the measures at day 1, week 4, month 3 and month 6 of intervention*

### Participant timeline

The participant timeline is presented in Table 1.

**Table 1:** Participant schedule of events

Assessment	Remote screening assessment day 14	Study time point					
		Baseline assessment day 1 (remotely)	IIET visit (within 7 days of Baseline visit)	Follow up telephone call — IE arm only (within 7 days of IIET visit)	Assessment 2 day 28–33 (remotely)	Assessment 3 month 3 (± 7 days) (remotely)	Assessment 4 month 6 (± 7 days) (remotely)
Medical history	X	X					
Concomitant medication	X	X		X	X	X	X
Consent	X						
Observed blood pressure and heart rate		X			X	X	X
IE ability test	X						
Incremental IE test and IE programme provided (for those randomised to intervention arm only)			X				
AE review				X	X	X	X
Diet questionnaire		X			X	X	X
Exercise questionnaire		X			X	X	X
Quality of life questionnaire		X			X	X	X
Collection of IE exercise diary (for those randomised to intervention arm only)				X	X	X	X
Collection of home blood pressure and heart rate readings	X (between day 7 and day 1)	X		X	X	X	X
Health resource use questionnaire					X	X	X
IE experience questionnaire					X		

### Sample size

Review of current literature revealed few IE studies in a hypertensive population. These studies were small (*n* < 25), conducted under different conditions to the proposed study, and showed low precision and large variability in estimates of the standard deviation (SD). A sample size of 100 participants, 50 per arm, will be used in the study. Allowing for 20% attrition and 6.5% incomplete data, 74 participants (37 in each arm) will have completed change measures at 4 weeks. This is in line with the recommended sample size of 70 to estimate key parameters from external pilot RCTs [62]. A sample size of 74 produces a two-sided 95% confidence interval with a width of 1.33 when the standard deviation is 4. This estimate of 4 has been taken from a previous study (*N* = 24) [10]. The sample size confidence interval has been calculated using Pass11 software (PASS 11. NCSS, LLC. Kaysville, UT, USA, www.ncss.com).

### Recruitment

Participants will be identified opportunistically through patient database searches and invited to participate. The study will also be advertised with ethically approved advertising materials, in participating GP practices, with electronic adverts on their websites, social media and newsletters as well as by text message to potential participants.

## Assignment of interventions: allocation

### Sequence generation

Random permuted blocks will be used within stratification (of site and age), ensuring that treatments are balanced at the end of every strata block. There are two stratification groups for age; these are age 18–49 years and ≥ 50 years. It is anticipated that between two to six primary care sites in South East England will be included in the study.

### Concealment mechanism

Participants will be allocated to either the control or isometric exercise arm using a third-party supplier of randomisation services [63]. This internet-based service allows investigators to randomise patients from anywhere in the world through a web browser.

### Implementation

The investigators will implement the allocation created by the online randomisation software [63].

## Assignment of interventions: blinding

This feasibility study was intended to replicate real life NHS practice where patients would be fully aware of any medical intervention prescribed by their healthcare professional. Therefore, the healthcare professionals (delivering the exercise intervention) and the participants/patients (performing the exercise) were not blinded to the allocated lifestyle intervention arms.

### Data collection and management

#### Plans for assessment and collection of outcomes

The success of intervention delivery by healthcare professionals in a primary NHS healthcare settings will be determined using both qualitative data (*remote*) and the heart rate data recorded by the participants at the end of each isometric wall squat bout x4 per session over the 3 sessions completed in the first week using a wireless heart rate monitor and chest strap (Sigma PC 15.11, Neustadt/Weinstraße, Germany). The average HR calculated from the first 3 sessions will then be compared against the individuals’ target training HR (calculated as 95% of HR_peak_ measured during the individuals incremental isometric exercise test IIET) with an acceptable target heart rate range (THRR) of 76–111% of heart rate peak [60]. Perceptions of intervention delivery by healthcare professionals will be explored in qualitative focus groups (*remote*). The focus group questions for both healthcare professionals and participants were guided by the work of O’Cathain et al. [64] on the use of qualitative research in feasibility studies for randomised controlled trials. The groups will discuss the intervention content and delivery and the trial design, conduct and processes. For professionals, the core questions will be the following: What are your views on how the isometric exercise was implemented? Is the study design acceptable? How did the planned recruitment practices work in practice? Are the proposed process and outcome measures valid for this group of service users?

Any change in BP following the IE intervention will be determined using the *home blood pressure data* (measured using an automated upper arm blood pressure monitor [Omron M3 Intellisense, Kyoto, Japan] at weeks 4, 12, and 24) recorded in the participants diaries and comparing this against their *observed* baseline (recorded at day 1). This data will also be used to estimate BP variance by calculating systolic BP change from baseline at each endpoint. An estimate of the difference in change from baseline between the isometric exercise and control group will be calculated with 80% and 95% confidence intervals.

Accuracy of IE protocol delivery will be determined using data from three fidelity assessments; the first being healthcare professional competency checks administered during their isometric exercise prescription training; the second being expert observation/evaluation of at least the first IIET delivered by each HCP; and then finally, by third party examination of the first week of heart rate data recorded in the study diaries of each HCP’s specific participants, with average HR calculated from the first 3 sessions being compared against the individuals target training HR as described above.

Execution of the IE training protocol in the home will be assessed for each individual participant at the end of the first week by checking to see if their mean training heart rate per week falls within target heart rate range/reference interval (76–111%) *data for weeks 4, 12 and 24 will now be assessed at the end of the 6-month training period*. An estimate of the short- (4 week) and medium-term (3 and 6 month) adherence rates to IE training will be based upon the data collected from participant diaries to calculate the proportion of participants completing ≥ two thirds of all IE sessions (12, 36 and 72) at each time point respectively. Patient recruitment and participant attrition rates will be calculated based upon the average number of participants recruited per week over the 7 month recruitment period and the number of withdrawals from the study once the last follow-up call to the last participant has been made.

Participant experiences of IE will be assessed through a quantitative online survey conducted at week 4. All participants receiving the IE intervention will be invited to take part in one of two focus groups (*remote*) to draw out respondents’ attitudes, feelings, beliefs, and experiences regarding the intervention [65]. The core focus group questions for participants were also directed by the guidelines for using qualitative research in feasibility studies [64]. What are your views on how you applied the isometric exercise intervention? What are your thoughts on how the study was conducted? What do you think are important outcomes to consider in a full trial? *This will also be used as an opportunity to explore possible negative effects of COVID-19 on recruitment rates and participation*. These groups will be held at month 4 and month 8 of the recruitment period with 6–8 participants in each group. One focus group will also be undertaken with healthcare professionals involved in the intervention delivery at the end of the recruitment period to explore views and experiences of the IE intervention. Lay and professional members of the research team will co-produce the topic guide and co-facilitate the focus groups. The focus groups are proposed to last for between 60 and 90 min. The groups will be digitally recorded and transcribed. Telephone interviews (*n* = 5–10) will be conducted with stakeholders from GP practices which are not recruitment sites and not involved in the intervention delivery to explore the willingness of GPs and primary care healthcare professionals to consider IE as a viable treatment option for patients, including barriers and facilitators for delivering and integrating this within an NHS care pathway for hypertension.

The economic evaluation of delivering the IE intervention will be calculated once the last follow-up call has been made to the last participant and will be estimated from Healthcare resource use data and quality-adjusted life years (QALYs). Since QALYs are the primary outcome of the economic evaluation, utility values will be obtained from patients’ responses to each of the EQ-5D-5L at the beginning of the intervention and at week 4 and months 3 and 6 after the intervention [66, 67].

New insight into the accuracy of home BP measurements to monitor changes in BP will be based upon the BP data (observed and home readings) recorded (at baseline assessment, day 1; assessment 2, week 4; assessment 3, month 3; and assessment 4, month 6) in the participant diaries, along with expert evaluation of the participants ability to carry out the measure.

#### Plans to promote participant retention and complete follow-up

Participant adherence will be measured as outcome data including the number of IE sessions completed. Completion of at least 8 of 12 sessions between baseline and the 4 week timepoint will be deemed adherence to the intervention. The percentage of completed IE sessions that meet the required target HR threshold will be calculated. The percentage of participants that deviate from protocol will be recorded to assess fidelity to the IE programme. The rate of healthcare professionals that pass the competency assessment after the half-day training session will be calculated.

#### Data management

Data entered directly into paper case report forms is considered as source data, additional source documentation includes participant study diaries, online questionnaires and focus group/interview audio recordings and transcripts. Data from case report forms and participant diaries will be entered manually into the database allowing for data monitoring and cleansing. Questionnaire data will be received and directly accessible online by only research team members who have the appropriate access. Case report forms will be shared with the coordinating centre via password secured email and a copy retained securely at site. A unique code will be produced for each participant and used on all corresponding documentation and files, to ensure anonymity.

#### Confidentiality

Only anonymised data will be shared with the coordinating centre for analysis. Electronic files with personal information will be password protected and stored on the university partner networks in folders that can only be accessed by the research team. Access to the data collected during the project (including any participant personal data) will be restricted to the research team, and data will not be shared with anyone else. Personal information that may enable the service user to be identified will be removed from interview and focus group transcripts.

Any personal data will be destroyed on completion of the project. The coded data will be stored for five years following the completion of the study, when it will be destroyed.

## Statistical methods

### Statistical methods for primary and secondary outcomes

#### Quantitative data analysis

Descriptive statistics will be used to assess primary and secondary process outcomes such as recruitment rates, adherence rates and completeness of data. Exercise adherence will be compared with outcomes to inform compliance criteria in the full study. In a definitive study, the primary outcome — change from baseline in systolic BP — will be analysed using analysis of covariance (ANCOVA), with a fixed treatment effect allowing adjustments for baseline values, centre, sex and age. This model will be used to estimate differences between the arms and confidence intervals from the feasibility study. Eighty percent and 95% confidence intervals will be calculated. Data from the IE experience questionnaires will be transferred to Stata/IC version 16 and analysed using descriptive statistics.

#### Qualitative data analysis

Thematic analysis of focus group/interview transcripts will be carried out using Braun and Clarke’s [68] six stage model using NVivo version 11 qualitative data analysis software (QSR International Pty Ltd. [2015] NVivo, https://www.qsrinternational.com/nvivo-qualitative-data-analysis-software/home). Drawing on Sweeney et al.’s [69] notion that the service user researcher unique perspective should be preserved rather than subsumed, the process will involve multiple members (including public co-applicants) of the project team. Inductive thematic analysis of focus group/interview transcripts will be carried out using NVivo version 11 (QSR International Pty Ltd. [2015] NVivo, https://www.qsrinternational.com/nvivo-qualitative-data-analysis-software/home). The process will involve reading and re-reading the transcripts and noting down initial ideas. Then the transcripts will be coded. Data extracts will be collated within each code and then codes ordered into potential themes. Subsequently, these themes will then be reviewed and refined. Ongoing analysis will refine the specifics of each theme and identify any themes which have not previously been recognised. Deviant case analysis will be used to ensure that perspectives that diverged from dominant trends are not overlooked.

### Methods in analysis to handle protocol non-adherence and any statistical methods to handle missing data

Missing values will be estimated using a combination of last observation carry forward and baseline observation carried forward (where only baseline data are available) in the secondary analysis of intention to treat.

### Plans to give access to the full protocol, participant level-data and statistical code

Anonymised data will be made available to researchers at universities, NHS organisations or other healthcare providers where the sharing of data has a clear defined purpose and its use will be of benefit to wider society.

## Oversight and monitoring

### Composition of the coordinating centre and trial steering committee

Delivery of the project is a collaboration between the Section of Sport, Exercise & Rehabilitation Sciences, Canterbury Christ Church University (CCCU), the Centre for Health Services Studies, University of Kent (UKC) and East Kent Hospitals University Foundation Trust (EKHUFT).

Two groups were established to provide appropriate oversight of the study and ensure this is maintained from various perspectives, the Study Steering Committee (SSC) and the Project Management Group (PMG).

The SSC is comprised of at least 75% independent members and includes the project co-leads, an independent statistician, a health economist, a clinician with expertise in hypertension/clinical trials/primary care, an exercise specialist and two independent lay members. The SSC meets tri-monthly to critically oversee progress, outputs, deliverables and governance of the project; have oversight of delivery of the study on behalf of the funder to ensure achievement of study objectives within agreed timelines; ensure the protection of rights and safety of study participants; regularly review of ongoing project data; and periodically review of safety data to ensure patient safety throughout [70]. In doing so, the SSC reports directly to the funder. The SSC will consider the need for any interim analysis based on reports received and may consider data emerging from other related studies and make recommendations for this study based on these. The SSC will also consider whether further time or funding is required for any aspect of the study and advise where best this may be obtained.

The PMG is comprised of 2 co-chief investigators, 4 co-applicants (including the project manager), 1 statistician, 1 health economist, 2 public co-applicants, 1 research facilitator and 1 study coordinator [70]. The members of PMG were responsible for the elaboration of this protocol and meets fortnightly with the purpose of maintaining clear oversight of study delivery according to the protocol, original grant application and subsequent changes; assessing the progress of the study and identifying any barriers to completion; agreeing mitigation plans and actions for any barriers to study completion, e.g. the current Covid-19 pandemic; providing advice to the Chief Investigators; ensuring the protection of rights and safety of study participants; and reporting on study progress to the SSC and funder.

The organizational study chart will include the sponsor EKHUFT, the SSC, the PMG, the two research units; UKC as lead on Qualitative data (health economics, focus group, interviews, patient participant involvement etc.) and CCCU as lead on quantitative data (intervention delivery, fidelity and patient outcomes etc.), the clinical research lead and the investigators at each primary care recruitment sites in SE England.

### Composition of the data monitoring committee, its role and reporting structure

A data monitoring committee is not needed for this study because the intervention is non-invasive with minimal risk of harm.

### Adverse event reporting and harms

An adverse event (AE) is any untoward medical occurrence in a study participant. A serious adverse event (SAE) is any untoward medical occurrence that: results in death, is life-threatening, requires inpatient hospitalisation or prolongation of existing hospitalisation, results in persistent or significantly disability/incapacity or consists of a congenital anomaly or birth defect. Other ‘important medical events’ may also be considered serious if they jeopardise the participant or require an intervention to prevent one of the above consequences.

Only AEs resulting (definitely or probably) from study procedures or the intervention will be collected by the research team and recorded in the participant’s medical records. These may be volunteered by the participant or discovered by the investigator or healthcare professional conducting study visits/follow-up telephone calls. They should be documented throughout the entirety of the study from randomisation until month 6 of intervention. Each AE will be evaluated by the Principal Investigator (PI) for seriousness, causality and expectedness.

A list of potential isometric exercise specific AEs was provided to the HCPs/investigators.

All SAEs will be recorded within 24 h of knowledge of the event. The SAE will be evaluated by the PI and when necessary, in collaboration with the Chief investigator (CI) (highly experienced renal consultant). The CI will report all SAEs to the Sponsor and the Research Ethics Committee within the conditions of ethical approval. The Study Steering Committee will periodically review safety data to ensure patient safety throughout.

### Frequency and plans for auditing trial conduct

Regular monitoring will be performed by the Study Coordinator to evaluate compliance with the protocol. Monitoring will verify that the study is conducted, and data are generated, documented and reported in compliance with the protocol and the applicable regulatory and national policy requirements [70]. Any data issues will be addressed by raising data queries for GP sites to resolve where possible, following this on-site monitoring will be undertaken. Direct access will be granted to authorised representatives from the Sponsor, host institution and the regulatory authorities to permit trial-related monitoring, audits and inspections - in line with participant consent.

### Plans for communicating important protocol amendments to relevant parties (e.g. trial participants, ethical committees)

Any substantial amendments to the protocol or other study documents may require review and approval by the Research Ethics Committee (REC) before the changes can be implemented to the study. Where amendments are required, NHS HRA and REC procedures will be followed, i.e. any amendment will be shared directly via email with participating sites. Sites will be asked to confirm receipt of the amended documents.

## Discussion

Systematic isometric wall squat exercise, which employs the large muscle mass of the quadriceps, has been shown to be an effective means of lowering resting BP. In a recent relatively small-scale study, Taylor et al. [10] showed that the magnitude of BP reduction following 4 weeks of isometric wall squat exercise in unmedicated high normal participants was greater than when the same intervention was completed by those with BP in the normal range [9]. It was postulated that this IE intervention may exert a greater antihypertensive effect in patients with more severe hypertension such as those diagnosed with stage 1 hypertension. The clinically significant BP reduction observed was also greater than the average BP reduction achieved with a single, standard dose anti-hypertensive drug [71] and is associated with reduced cardiovascular mortality [5]. However, before the efficacy of this novel intervention to treat stage 1 hypertension can be investigated in any large-scale randomised controlled trial, it is first necessary to ascertain if it can be delivered and carried out as intended in (as ultimately envisioned) an NHS primary care setting. If it can, and patient adherence is found to be equal or better than that documented for other forms of lifestyle invention and resting BP is shown to either decrease or stay the same following the IE intervention, then the findings of this feasibility study will support the viability of IE as both a prophylactic and alternative treatment option for thousands of NHS patients wishing to avoid antihypertensive drug therapy. Importantly, the results of this study will determine uncertain parameters needed to design a substantive efficacy study, including the variance in BP change needed for sample size calculation, the ability to deliver the intervention as intended in primary care, recruitment rates, and participant acceptability and compliance. Moreover, the economic analysis in this feasibility study will determine the acceptability of resource use and utility measures in this setting, establish the cost of the IE intervention programme and conduct some preliminary modelling to explore potential cost effectiveness, prior to a full economic evaluation of the IE intervention programme in the planned definitive trial. The follow-on study will be a randomised, controlled efficacy study pending additional funding. This study will determine uncertain parameters needed to design a substantive trial; variance of the primary outcome measure needed for sample size calculation, ability to deliver the intervention as intended in primary care, recruitment rates and participant acceptability and compliance.

Whilst the advent of Covid-19 has presented a significant challenge, primarily in terms of reliably delivering and evaluating a novel exercise training intervention remotely, it has also provided a valuable opportunity to evaluate alternate methodological approaches/new protocols that may not otherwise have occurred, e.g. the feasibility of using observed patient home BP readings for remote monitoring as opposed to clinic measurements. Since the current pandemic shows little sign of abating, this additional information might help to inform the research design of similar studies planned for delivery during these exceptional times.

## Supplementary Information

**Additional file 1.** EKHUFT leaflet SLA adapted (.pdf). This document displays the standard lifestyle advice information shared with participants.

## Data Availability

Anonymised data will be made available upon request, to researchers at universities, NHS organisations or other healthcare providers where the sharing of data has a clear defined purpose and its use will be of benefit to wider society.

## Abbreviations

BP: Blood pressure
IE: Isometric exercise
NHS: National Health Service
PHE: Public Health England
CKD-EPI: Chronic Kidney Disease Epidemiology Collaboration
MDRD: Modification of Diet in Renal Disease
QALYs: Quality-adjusted life years
AE: Adverse event
SAE: Serious adverse event
CCCU: Canterbury Christ Church University
UKC: University of Kent
EKHUFT: East Kent Hospitals University Foundation Trust
SSC: Study Steering Committee
PMG: Project Management Group
