# Supplementary material for: Feasibility study to assess the delivery of a novel isometric exercise intervention for people with stage 1 hypertension in the NHS: protocol for the IsoFIT-BP study including amendments to mitigate the risk of COVID-19
Source: Pilot Feasibility Stud. 2021 Oct 28;7:192. doi: 10.1186/s40814-021-00925-w (PMC8551940; doi:10.1186/s40814-021-00925-w)
Supplement: Supplementary file 1 — Additional file 1. EKHUFT leaflet SLA adapted (.pdf). This document displays the standard lifestyle advice information shared with participants. [file 40814_2021_925_MOESM1_ESM.pdf]

East Kent Hospitals University NHS Foundation Trust

High blood pressure (Hypertension)

Information for patients from the Department of Renal (Kidney) Medicine

This leaflet is not meant to replace the information discussed between you and your doctor, but can act as a starting point for such a discussion or as a useful reminder of the key points.

What is high blood pressure (hypertension) and what causes it?

High blood pressure is one of the most common and preventable causes of premature heart disease, kidney disease, and stroke in the UK. About one in four adults and more than half of those over 60 years old are affected.

The cause of high blood pressure is not fully understood in most of those affected, who are generally described as having 'essential hypertension'.

A number of different factors are known to be important. These include:

- excessive retention of salt in the body by the kidneys
- reduced blood flow in the kidneys, partly due to increased activity of nerves involved in 'stress' responses (the sympathetic nervous system)
- increased activity in a complex system of hormones (known as the 'renin-angiotensin-aldosterone' system).

How is blood pressure measured?

Blood pressure is usually measured using a device called a sphygmomanometer or 'blood pressure monitor' where a cuff is applied to the upper arm over the brachial artery.

When measuring blood pressure, two readings are taken at the same time. The upper reading (systolic) reflects the pressure generated within the arteries when the heart is contracting. The lower reading (diastolic) reflects the pressure that remains in the artery when the heart is relaxing and filling. Blood pressure is therefore often presented as 'systolic/diastolic' with units 'millimeters of mercury' or 'mmHg' for short. For example: 140/85 mmHg, where 140 is the systolic, and 85 is the diastolic blood pressure.

Although both systolic and diastolic readings have significance, most guidelines and targets for 'optimal' blood pressure control now focus on systolic blood pressure (the upper reading).

How can I measure my blood pressure at home?

Home blood pressure monitoring is recommended for people with hypertension by all major guidelines (for example the National Institute of Health and Care Excellence (NICE)). Guidance on how to measure your blood pressure at home can be found at www.bloodpressureuk.org/BloodPressureandyou/Homemonitoring/Howtomeasure. In general, when you are asked to collect home blood pressure readings (for example before your clinic appointment) we will ask you to take measurements over five days, with two to three readings in the morning and evening of each of those days.

The British and Irish Hypertension Society (BIHS) publishes a list of validated blood pressure monitors that you can use at home on their web site bihsoc.org/bp-monitors/for-home-use/. More information on home blood pressure monitoring can be found at www.bloodpressureuk.org/BloodPressureandyou/Homemonitoring.

What is the 'white coat effect'?

Around one quarter of people have the 'white coat effect' where their blood pressure readings are higher at their GP's surgery or hospital clinic than they are at home. For these people, home or automated wearable device ('ambulatory') blood pressure measurements may be very helpful.

How is high blood pressure treated?

The standard treatment for high blood pressure in the UK consists of lifestyle changes such as reducing salt in the diet, increasing exercise, and losing weight where appropriate, plus blood pressure lowering medications.

Lifestyle changes that are recommended for people with high blood pressure include the following.

- **Salt intake**. The average salt intake in the UK is nine grams per day. The recommended daily salt intake for an adult is five to six grams per day. Reducing the amount of salt in your food lowers blood pressure, and makes blood pressure lowering medications more effective. Although stopping adding salt to your food when cooking or at the table is important, 80% of the salt we eat is already present in food when we buy it – so called ‘hidden salt’. Understanding food labelling can help you see where these hidden sources of salt are found.

- **Healthy diet**. You should follow a diet that is rich in fresh fruit and vegetables, and low in saturated fat.

- **Weight**. You should try to maintain a body mass index between 20 and 25 kg/m2. Those with BMI greater than 30 kg/m2 and high blood pressure should be definitely try to lose weight to achieve a BMI of 30 kg/m2 or below. See BMI chart at the end of this leaflet.

- **Alcohol consumption**. You should adhere to the current UK limit of less than 14 units per week. If you do regularly drink as much as 14 units in a week try to spread your drinking evenly over three days or more. One unit of alcohol is equivalent to half-pint of average strength beer; a small glass of wine (125 ml); or a single pub measure of spirits. Remember that stronger beers (such as continental lager) and larger glasses of wine (175 ml or 250 ml) will contain more units.

- **Exercise**. You should try to exercise at a level that makes you breathless for at least 30 minutes three times a week, although more is recommended if possible. A variety of different types of exercise – aerobic/endurance (for example running) and resistance (for example weights) - both appear to be equally helpful in reducing blood pressure.

For more information on lifestyle modification visit the Blood Pressure UK website
www.bloodpressureuk.org/BloodPressureandyou/Yourlifestyle

Medicines for high blood pressure are made up of three common classes

- “Renin-angiotensin system” blocking drugs: these block various parts of a complex hormone system called the “renin-angiotensin system”. Examples include: ACE inhibitors (end in –pril) such as ramipril; angiotensin receptor blockers (end in –sartan) such as losartan or irbesartan; renin inhibitors such as aliskiren.

- Vasodilators: these directly relax muscles in the blood vessel wall. Examples include: calcium channel blockers such as amlodipine; alpha-blockers such as doxazosin.

- Diuretics or “water pills”: these encourage the kidney to excrete salt and water. Examples include: bendroflumethiazide, indapamide, frusemide, spironolactone.

For more information on blood pressure lowering medicines visit the Blood Pressure UK website
www.bloodpressureuk.org/BloodPressureandyou/Medicines

My blood pressure is not controlled – why is that?
Despite treatment, in about half of people taking medication, their blood pressure will be higher than desirable. There are a number of possible reasons for this.

- **Inadequate numbers of blood pressure medications**: many people need three or more different types of blood pressure tablet.

- **Inadequate doses of blood pressure medications**: although some people will not be able to tolerate higher doses of some tablets due to the side effects, it is quite common for less-than maximal doses of medication to be prescribed without a good reason.

- Not taking medications regularly or in amounts suggested by your doctor.

- Not enough changes to your lifestyle.

- The “white coat effect”.

- **“Secondary” hypertension**: underlying causes such as over-production of certain hormones (for example aldosterone) or kidney artery narrowing are found in five to 10 our of every 100 people with uncontrolled blood pressure.

If you or your doctor think any of the above apply to you, this can be discussed at your clinic visits.

What is resistant hypertension?
Resistant hypertension is generally means blood pressure that remains uncontrolled in someone who is taking at least three or four blood pressure lowering medications at an adequate dose, who has made appropriate changes to their lifestyle (for example salt intake, weight loss), and who has been investigated for underlying causes of high blood pressure. People with resistant hypertension also need to have the ‘white coat effect’ excluded by ambulatory or home blood pressure monitoring.

People with resistant hypertension are more likely to suffer heart disease or stroke, compared to people with controlled blood pressure. Treatment options for people with resistant hypertension include additional blood pressure lowering medications – for example, spironolactone or amiloride – or sometimes enrolment into clinical trials of novel treatments such as renal denervation or other device based therapy.

Where can I get more information?

- Blood Pressure UK www.bloodpressureuk.org/Home
  (Section on salt: www.bloodpressureuk.org/BloodPressureandyou/Yourlifestyle/Eatingwell/Salt)

- British Heart Foundation www.bhf.org.uk/heart-health/prevention.aspx

Thank you for taking the time to read this information leaflet. If you have any further questions please speak to your GP.

Body Mass Index Chart

Body Mass Index (BMI) is calculated by dividing your weight (in kilograms) by your height (in metres) squared.

- A BMI less than 18.5 kg/m2 is underweight.
- A BMI between 18.5 and 24.9 kg/m2 is a healthy weight.
- A BMI between 25 and 29.9 kg/m2 is overweight.
- A BMI of 30 kg/m2 or higher is obese (well above the healthy weight range for your height).
- A BMI of 40 kg/m2 or higher is very obese.

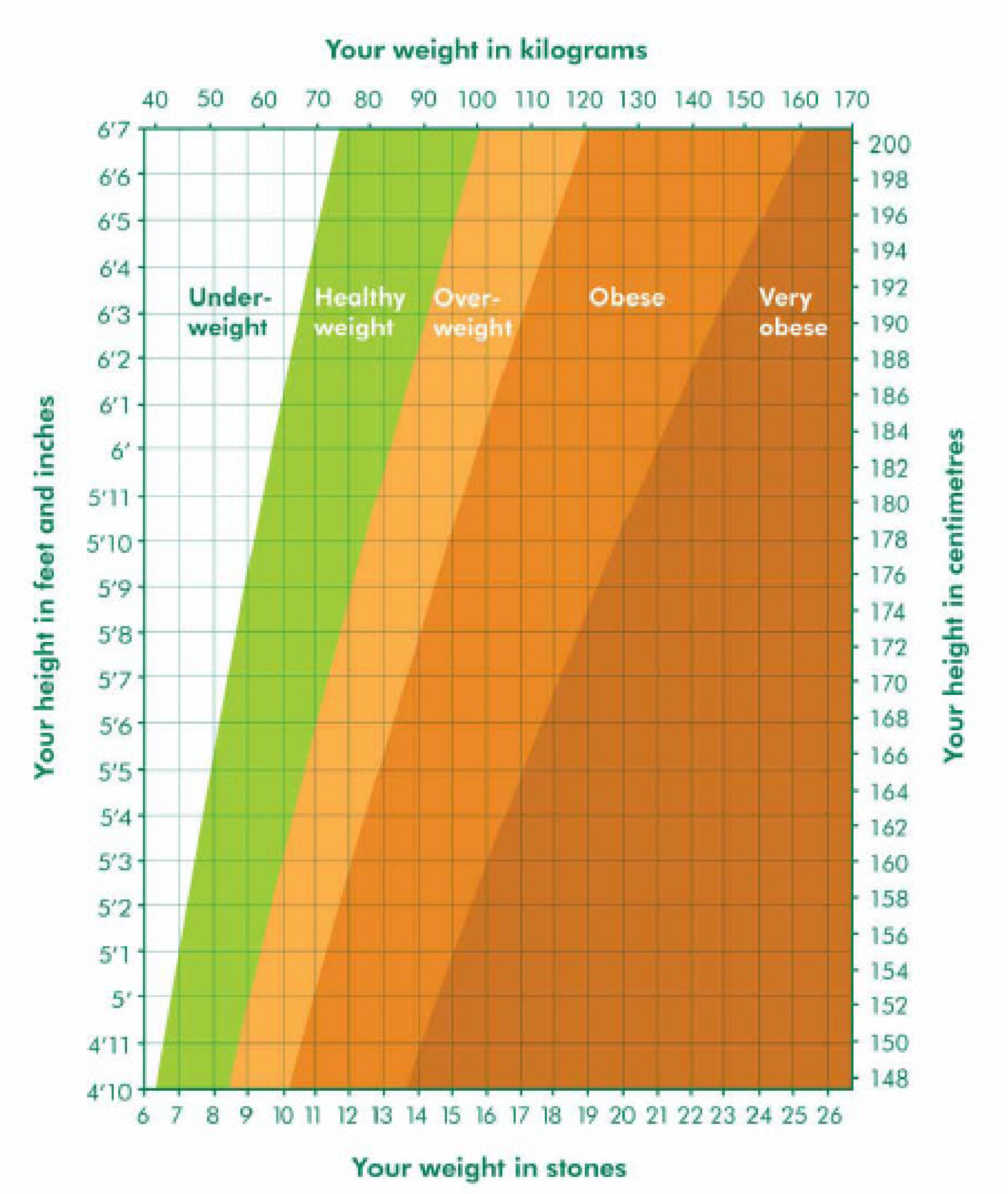

This leaflet has been produced with and for patients

If you would like this information in **another language, audio, Braille, Easy Read, or large print** please ask a member of staff.

Any complaints, comments, concerns, or compliments please speak to your doctor or nurse, or contact the Patient Advice and Liaison Service (PALS) on 01227 783145, or email ekh-tr.pals@nhs.net

Further patient leaflets are available via the East Kent Hospitals web site www.ekhuft.nhs.uk/patientinformation

Information produced by the Department of Renal (Kidney) Medicine
Date reviewed: May 2020 Next review date: September 2022 Web 240
